# Prehospital 12-Lead ECG Teletransmission in Acute Coronary Syndromes: Diagnostic, Prognostic, and Systems-of-Care Implications

**DOI:** 10.3390/jcm15155840

**Published:** 2026-07-26

**Authors:** Ernest Tomczak, Lukasz Gawinski, Karol Matewski, Remigiusz Kozlowski

**Affiliations:** 1 Department of Cardiology, Invasive Cardiology and Electrophysiology, Regional Specialist Hospital, 86-300 Grudziadz, Poland; mrgawinski@gmail.com (L.G.); karmat787@gmail.com (K.M.); 2 Center of Security Technologies in Logistics, Faculty of Management, University of Lodz, 90-237 Lodz, Poland; remigiusz.kozlowski@uni.lodz.pl

**Keywords:** ECG teletransmission, prehospital ECG, ST-elevation myocardial infarction, acute coronary syndrome, organizational implications, door-to-device time, first medical contact-to-device time, cath lab activation

## Abstract

**Background/Objectives:** Acute coronary syndrome (ACS) remains one of the leading causes of cardiovascular mortality worldwide. Rapid diagnosis and timely initiation of reperfusion therapy are crucial for improving patient outcomes. The aim of this narrative review was to summarize current evidence on prehospital 12-lead ECG teletransmission in the diagnosis and management of ACS and to evaluate its impact on systems of care and clinical outcomes. **Methods:** A narrative literature review was conducted using the PubMed database. The literature search was completed on 15 May 2026 and included publications from 1998 onwards. The review included studies evaluating prehospital ECG teletransmission, ST elevation myocardial infarction (STEMI) networks, interpretation models, treatment delays, and organizational and logistics aspects of ECG teletransmission systems. Current guidelines from the European Society of Cardiology and the 2025 ACC/AHA/ACEP/NAEMSP/SCAI guidelines were also included. **Results:** Available evidence indicates that prehospital ECG teletransmission enables earlier STEMI diagnosis, direct referral to primary coronary intervention (PCI)-capable centers, and earlier activation of catheterization laboratories. Multiple observational studies and meta-analyses have demonstrated reductions in first medical contact-to-device and door-to-device times associated with teletransmission systems. Limitations include interpretation variability, false-positive catheterization laboratory activations, and infrastructure-related barriers. **Conclusions:** ECG teletransmission is an essential component of contemporary regional STEMI care systems and contributes to improved organization of care and shorter reperfusion delays. Further development and prospective validation of artificial intelligence-assisted ECG interpretation and integrated telemedicine systems may enhance diagnostic accuracy and the effectiveness of prehospital cardiac care.

## 1. Introduction

Cardiovascular diseases remain the leading cause of death in member countries of the European Society of Cardiology (ESC), accounting for approximately 2.2 million deaths among women and over 1.9 million deaths among men annually, which corresponds to 45% and 39% of all deaths in females and males, respectively [1]. These figures highlight the substantial burden of cardiovascular diseases on public health and healthcare systems, with far-reaching economic and societal consequences. They not only impose a substantial burden in terms of mortality but also have long-term effects on patients’ quality of life, disability, treatment costs, and social productivity. In 2019, approximately 5.8 million new cases of ischemic heart disease were reported across the 57 member countries of the ESC, illustrating the large number of patients requiring intensive cardiac care [2]. Acute coronary syndrome (ACS)—which includes ST-segment elevation myocardial infarction (STEMI) and non-ST-segment elevation myocardial infarction (NSTEMI)—constitutes a significant portion of patients with coronary artery disease. In many European countries, the estimated incidence of STEMI ranges from approximately 40 to over 140 cases per 100,000 inhabitants per year. Analyses of mortality trends in European Union countries show that between 2012 and 2020—despite a decreasing trend—a total of over 1.7 million people died from ACS [3]. In addition to the symptoms typical of ACS, the diagnosis of myocardial infarction is based on a 12-lead electrocardiogram (ECG). Diagnostically, it is crucial to demonstrate dynamic changes in cardiac troponin levels, along with at least one indicator of ischemia, such as characteristic changes in the ECG or typical clinical symptoms. In clinical practice, it is important to perform an ECG promptly (preferably within 10 min of patient contact) and simultaneously assess cardiac markers; this is essential for early classification into STEMI and NSTEMI and for selecting the appropriate therapeutic strategy [2]. Despite these established diagnostic criteria, the early diagnosis of myocardial infarction remains clinically challenging. The initial ECG may be normal during the early stages of myocardial ischemia, and ECG interpretation may be challenging in several clinical situations. These include left bundle branch block (LBBB), ventricular pacing, left ventricular hypertrophy, previous myocardial infarction with residual scarring, and several non-ischemic conditions associated with ST-segment abnormalities. Contemporary myocardial infarction guidelines recommend the use of ECG teletransmission systems to facilitate diagnosis and enable the earlier implementation of appropriate treatment [4]. EMS ambulances are equipped with portable ECG devices as standard (most often as part of a mobile defibrillator), which allows the first responders to perform an ECG at the scene (e.g., at the patient’s home or in a public place) [5]. The use of telematic systems enables healthcare professionals to transport patients with STEMI directly to PCI-capable (primary coronary intervention-capable) centers, bypassing hospitals without interventional cardiology facilities (non-PCI centers). The review included studies evaluating prehospital ECG teletransmission, ST elevation myocardial infarction (STEMI) networks, interpretation models, treatment delays, and organizational and logistics aspects of telecardiology systems [2]. The aim of this narrative review was to summarize the current evidence regarding the use of 12-lead electrocardiogram (ECG) teletransmission in the diagnosis and treatment of ACS and to evaluate its impact on treatment organization and clinical and organizational outcomes.

Rather than focusing solely on the diagnostic performance of ECG teletransmission or its effect on reperfusion delays, this review examines teletransmission as an integral component of contemporary STEMI systems of care, emphasizing the organizational, logistical, and clinical factors that determine its effectiveness in routine practice.

To facilitate clinical interpretation, the review follows the sequence of the prehospital management pathway, beginning with ECG acquisition and transmission, followed by ECG interpretation, activation of STEMI systems of care, organizational aspects of reperfusion therapy, applications beyond STEMI, current limitations, and future developments.

## 2. Materials and Methods

This article provides a narrative review of the literature on the use of 12-lead ECG teletransmission in the diagnosis and treatment of ACS, with a particular focus on STEMI and NSTEMI.

The literature review was conducted in the PubMed database, covering publications from 1998 to 2025, with a particular emphasis on studies published after 2010.

The literature search was conducted in the PubMed database and covered publications from 1998 through May 2026, with particular emphasis on studies published after 2010. The search strategy combined the following keywords: “ECG teletransmission”, “prehospital ECG”, “STEMI”, “NSTEMI”, “acute coronary syndrome”, “telemedicine”, “artificial intelligence”, “AI”, “electrocardiography”, “electrocardiography interpretation”, “FMC-to-device time”, and “catheterization laboratory activation”. The literature search was last updated on 15 May 2026.

A total of 48 publications were screened, of which 30 met the predefined eligibility criteria and were selected for inclusion based on their relevance to the objectives of this review. Preference was given to original studies, systematic reviews, meta-analyses, clinical guidelines, and key organizational reports concerning prehospital ECG teletransmission, STEMI systems of care, telemedicine in cardiology, and the emerging role of artificial intelligence in ECG interpretation.

The inclusion criteria comprised studies evaluating the clinical and organizational impact of ECG teletransmission, including its influence on diagnostic accuracy, treatment delays, catheterization laboratory activation, systems of care, and the implementation of artificial intelligence in prehospital ECG interpretation. Additional emphasis was placed on studies investigating the effect of ECG teletransmission on reducing the time from first medical contact to reperfusion therapy and on publications discussing future developments in telemedicine and digital cardiovascular care.

Exclusion criteria included publications older than 30 years, studies focused exclusively on non-ST-elevation myocardial infarction (NSTEMI), conference abstracts without full-text availability, and articles not directly related to the scope of prehospital ECG teletransmission.

The final selection of publications was based on their scientific relevance, methodological quality, and contribution to the clinical and organizational aspects of ECG teletransmission discussed in this review.

Due to the nature of the narrative review, the formal PRISMA methodology was not applied. The analysis was qualitative and synthetic, with an emphasis on clinical and organizational implications as well as future directions for the development of ECG teletransmission technology.

## 3. Definitions and Principles of ECG Teletransmission

Currently, no universally accepted definition of ECG teletransmission exists in the literature. Therefore, for the purposes of this review we adopted the following working definition: ECG teletransmission refers to the digital acquisition and remote transmission of a 12-lead electrocardiogram to a reference centre for expert interpretation, clinical decision-making, and activation of appropriate treatment pathways. It represents one of the core technological components of contemporary telecardiology systems.

Although the terms “ECG teletransmission” and “telecardiology” are sometimes used interchangeably, telecardiology is a broader concept encompassing a wide range of telemedicine applications in cardiovascular care, including ECG teletransmission, remote monitoring of implantable cardiac devices, and telemonitoring of patients with heart failure. The principal advantage of ECG teletransmission is its ability to facilitate rapid specialist consultation and support timely clinical decision-making in patients with suspected acute coronary syndromes. Effective implementation requires reliable ECG acquisition, secure telecommunications infrastructure, dedicated software, and appropriately trained healthcare professionals. To maximize its clinical effectiveness, ECG teletransmission should be integrated within well-organized regional systems of care with clearly defined referral pathways. One important limitation of telemedicine is the absence of direct patient contact, which may restrict clinical assessment because physical examination cannot be performed and important clinical information may not always be fully communicated by emergency medical services personnel.

This ensures that emergency medical teams are assigned to a specific catheterization laboratory, which in turn facilitates communication and the assessment of a patient’s need for urgent intervention and treatment. A separate issue, beyond the scope of this review, is the appropriateness of the use of ECG teletransmission systems by emergency medical teams [5].

## 4. Data Transmission Models in ECG Teletransmission Systems

In clinical practice, there are two basic models of data transmission: real-time transmission and the “store-and-forward” model. The main differences between store-and-forward and real-time transmission methods are summarized in Table 1.

1. Real-time ECG transmission involves the immediate transmission of the ECG recording during its acquisition or immediately after its completion, with minimal technological delay. It allows for continuous or nearly immediate signal transmission, the ability for a physician at the destination center to view the recording simultaneously, interactive consultation (e.g., by phone between the emergency team and a cardiologist), and the immediate initiation of therapeutic decisions (activation of the catheterization laboratory).
2. Store-and-forward transmission remains widely used in telemedicine applications such as outpatient ECG review, remote consultations, and data archiving. However, in the management of STEMI, real-time or near-real-time ECG transmission with immediate interpretation is the preferred model, as it enables rapid activation of regional reperfusion pathways and minimizes treatment delays.

**Figure 1:**
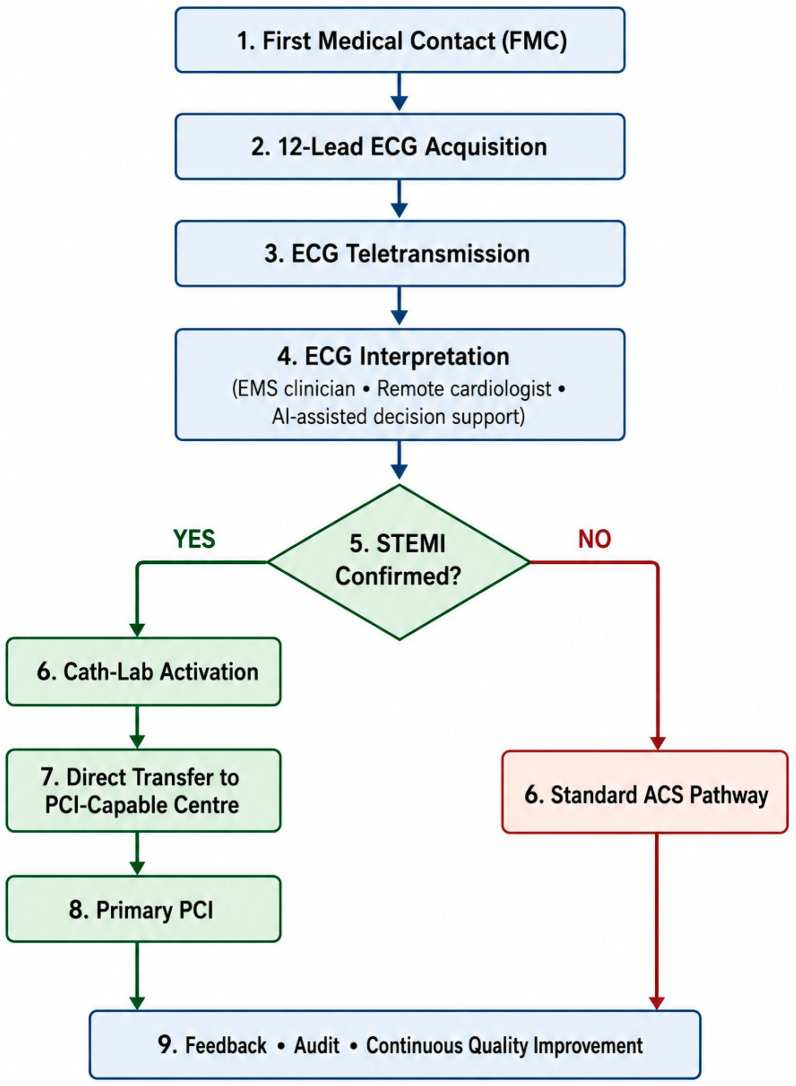
Prehospital ECG teletransmission pathway within regional STEMI system of care. Abbreviations: ACS—acute coronary syndrome; AI—artificial intelligence; EMS—emergency medical services; FMC—first medical contact; PCI—percutaneous coronary intervention; STEMI—ST-segment elevation myocardial infarction.

The overall workflow of prehospital ECG teletransmission within contemporary STEMI systems of care is illustrated in Figure 1.

## 5. Organizational Models of ECG Teletransmission Systems

The clinical benefits of ECG teletransmission are determined not only by the technology itself but also by the organization of the regional STEMI system in which it operates.

The implementation of 12-lead ECG teletransmission in the diagnosis of ACS is a component of the complex organizational structures of regional STEMI treatment systems. Current ESC guidelines emphasize that the effectiveness of reperfusion therapy depends not only on the availability of technology but, above all, on the organization of regional STEMI systems of care and the minimization of treatment delays. These guidelines also highlight the importance of organized regional STEMI networks, which standardize patient triage, optimize transport logistics, and facilitate timely reperfusion [2].

Four principal organizational models of ECG teletransmission can be distinguished. The first is the prehospital direct activation model, in which a 12-lead ECG acquired in the ambulance is transmitted to a PCI-capable referral center for expert interpretation. This enables confirmation of the diagnosis of STEMI and activation of the catheterization laboratory before the patient’s arrival, thereby shortening the time to reperfusion.

The ESC guidelines recommend performing and interpreting an ECG within 10 min of the first medical contact and direct transport to a PCI center if the time to reperfusion falls within the recommended time frame [2]. Registry studies and systematic reviews have shown that the model of prehospital ECG teletransmission is associated with a reduction in First Medical Contact (FMC) to device time and a reduction in in-hospital mortality [6,7].

Another model is the hub-and-spoke network model, which relies on the centralization of clinical decision-making at a referral center (hub), to which ECG recordings are transmitted from smaller units (spokes), such as ambulances or hospitals without PCI capabilities. The randomized DANAMI-2 trial demonstrated the superiority of transport to a center with PCI capabilities over fibrinolytic therapy in hospitals without PCI, which formed the basis for the development of regional STEMI networks [8].

The third model of logistics organization is the central consultation center model. In this variant, ECG interpretation takes place at a specialized telecardiology center serving multiple prehospital units. System analyses have shown that remote ECG interpretation by a cardiologist may reduce the risk of ECG misinterpretation and shorten the time to activation of the catheterization laboratory [7,9]. This model is particularly important in regions with limited access to specialists and in systems with a high diagnostic workload.

The fourth and final model—the interhospital transfer model—is of critical importance in the management of patients with ACS, as it enables the rapid transmission of ECG data between non-PCI hospitals and referral centers, facilitating prompt eligibility assessment for urgent angiography and transfer. Observational studies indicate that early teleconsultation and transfer for PCI improve treatment outcomes compared to delayed qualification [8,10]. ESC guidelines clearly state that reducing the time to reperfusion (total ischemic time) is a key prognostic factor in STEMI [2]. Therefore, the mere availability of ECG teletransmission technology is insufficient to improve outcomes unless it is fully integrated into coordinated STEMI systems of care that ensure rapid interpretation, direct PCI-center activation, and efficient patient transport [6,7].

## 6. Organization of the ECG Interpretation Process

The effectiveness of 12-lead ECG teletransmission depends not only on the quality of signal transmission, but above all on the interpretation model and the organization of the decision-making process. The ESC guidelines emphasize the need to perform and interpret an ECG within 10 min of the first medical contact [2]. Similar recommendations are included in the recent ACC/AHA/ACEP/NAEMSP/SCAI Guideline for the Management of Acute Coronary Syndromes, which emphasizes early prehospital ECG acquisition, prompt interpretation, and integration within regional systems of care [11]. In teletransmission systems, three main interpretation models are distinguished: assessment by prehospital personnel, remote interpretation by a cardiologist, and algorithm-assisted analysis. In many systems, the initial assessment of the ECG is performed by the emergency medical services (EMS) team (interpretation by prehospital personnel). It has been demonstrated that the use of prehospital ECG is associated with a reduction in time to reperfusion and improved organization of STEMI care [9]. Systematic reviews confirm that performing a 12-lead ECG in the prehospital setting and notifying the receiving facility in advance can help reduce system-wide delays and mortality [12]. At the same time, significant variability in interpretation has been demonstrated in the prehospital setting, which may influence triage decisions and eligibility for reperfusion [13]. This problem particularly affects patients with left bundle branch block (LBBB), pacemaker rhythm, and non-specific ST-segment changes. Therefore, remote interpretation by a cardiologist appears to be crucial. This model involves transmitting the ECG recording to a referral center, where a specialist performs the interpretation. A study by Sejersten et al. demonstrated that teletransmission of ECGs to a cardiologist enables direct referral of the patient to a PCI center and reduces treatment delays [6]. Analyses of regional systems indicate that the integration of teleconsultation into the organization of the STEMI network improves system efficiency and the quality of care [14,15]. Furthermore, one meta-analysis confirms that prehospital digital ECG teletransmission significantly reduces the time to revascularization and may lower mortality in STEMI [16]. Currently, numerous studies are underway to enhance the accuracy of algorithm-assisted ECG interpretation. Modern teletransmission systems increasingly utilize digital and cloud-based solutions that enable automatic ECG analysis [17]. Computer algorithms achieve high sensitivity in detecting STEMI; however, their limited specificity may increase the rate of false-positive catheterization laboratory activations [18].

The effectiveness of ECG interpretation in teletransmission systems is closely linked to the organization of regional STEMI care networks. The DANAMI-2 and PRAGUE studies demonstrated the superiority of well-organized transport systems to PCI centers over decentralized models [8,10]. Reducing total ischemia time remains a key prognostic factor, and achieving this requires both early ECG diagnosis and an efficient decision-making pathway leading to reperfusion [2,11]. Both the ESC and ACC/AHA guidelines emphasize that automated interpretation should support clinical decision-making, not replace it [2,11].

The principal clinical studies evaluating the impact of prehospital ECG teletransmission on diagnosis, treatment organization, and clinical outcomes are summarized in Table 2. Overall, the available evidence consistently demonstrates that prehospital ECG teletransmission reduces treatment delays, improves the organization of regional STEMI systems of care, achieves high diagnostic performance, and is associated with improved clinical outcomes, although the strength of evidence varies according to study design. To sustain these benefits and identify opportunities for further system optimization, continuous quality monitoring and regular audit are essential components of mature regional STEMI systems of care [14].

## 7. The Role of ECG Transmission in the Diagnosis of STEMI

The effectiveness of STEMI diagnosis in the prehospital setting should be analyzed from multiple perspectives, encompassing both classic diagnostic accuracy parameters (sensitivity, specificity, positive predictive value) and systemic effectiveness as measured by the reduction in reperfusion delays, the percentage of unjustified catheterization laboratory activations (false-positive activation), and the impact on mortality. A meta-analysis evaluating various models for interpreting prehospital ECGs (EMS personnel, remote interpretation, automated algorithms) demonstrated high sensitivity for the diagnosis of STEMI, with variable specificity depending on the interpretation model [19]. Models based on remote specialist consultation demonstrated higher positive predictive values compared to independent prehospital interpretation. Studies evaluating remote ECG interpretation by emergency medicine physicians confirmed high concordance of STEMI diagnoses with coronary angiography results, while simultaneously reducing unjustified activations of the catheterization laboratory [20]. However, it should be emphasized that, in contemporary emergency medical systems across most European countries, ambulance crews are predominantly staffed by paramedics rather than emergency physicians.

Analyses of systems using wireless transmission of 12-lead ECGs have shown that the implementation of teleconsultations reduces the rate of false catheterization laboratory activations while maintaining high diagnostic sensitivity [21]. A significant proportion of false-positive STEMI diagnoses remains a problem, and this is also one of the most frequently analyzed efficacy indicators. Observational studies have shown that misdiagnoses of STEMI most often result from the presence of conduction abnormalities (particularly LBBB), early repolarization, myocarditis, and recording artifacts [23]. Modifying the interpretation algorithms used in prehospital devices can significantly reduce the number of false-positive STEMI diagnoses without significantly compromising diagnostic sensitivity [24]. Recent studies indicate that artificial intelligence-based systems can improve the positive predictive value of prehospital STEMI diagnosis by reducing unjustified activations of the catheterization laboratory, while maintaining high sensitivity for detecting ST-segment elevation [22].

However, in line with previous analyses regarding computer-aided ECG interpretation, algorithms should be viewed as a tool to support clinical decision-making, not as a substitute for it [18]. Similar conclusions emerge from systematic reviews analyzing the impact of prehospital ECG and notification of the destination center on treatment outcomes [12].

In this context, diagnostic effectiveness is not merely a technical issue but has a direct impact on total ischemic time and, consequently, patient outcomes. A recent systematic review and meta-analysis including more than 67,000 patients demonstrated that prehospital interpretation of 12-lead ECGs for STEMI diagnosis has a high overall diagnostic performance, with a pooled sensitivity of 80% (95% CI 69–88%) and a pooled specificity of 97% (95% CI 94–98%) [19]. Diagnostic performance differed according to the ECG interpretation strategy. Interpretation by ambulance clinicians demonstrated the highest sensitivity (94%, 95% CI 80–98%) with a specificity of 94% (95% CI 74–99%), whereas computer-assisted interpretation achieved a sensitivity of 73% (95% CI 58–84%) and a specificity of 97% (95% CI 93–98%). ECG transmission with remote specialist interpretation was characterized by a sensitivity of 72% (95% CI 58–83%) and the highest specificity (98%, 95% CI 94–99%) [19]. The pooled estimates represent weighted summary estimates across all included studies, whereas the subgroup analyses describe the diagnostic performance of specific ECG interpretation strategies. From a clinical perspective, lower sensitivity increases the likelihood of false-negative STEMI diagnoses and delayed reperfusion, whereas lower specificity may increase unnecessary catheterization laboratory activations.

Studies evaluating ECG interpretation by emergency physicians have demonstrated high concordance between STEMI diagnoses and coronary angiography findings while reducing unnecessary catheterization laboratory activations [20]. Likewise, systems incorporating wireless ECG transmission with direct catheterization laboratory activation have improved the appropriateness of primary PCI activation while shortening treatment delays [6,21].

False-positive catheterization laboratory activation is an important indicator of STEMI network performance. Across studies included in the meta-analysis, false-positive activation rates ranged from approximately 11% to 16%, reflecting differences in ECG interpretation strategies and organizational models [19]. For example, in the wireless ECG transmission system evaluated by Tanguay et al., the false-positive catheterization laboratory activation rate was approximately 11%, representing the lower end of the range reported across the included studies [19,21].

As previously mentioned, observational studies analyzing the causes of misdiagnosed STEMI indicated that the most common causes of false activations were the following:

- LBBB;
- Early repolarization;
- Myocarditis;
- Recording artifacts, and incorrect electrode placement [21].

The implementation of modified interpretation algorithms in prehospital devices can significantly reduce the rate of false-positive diagnoses without significantly compromising sensitivity [24].

Another important limitation of prehospital STEMI diagnosis is the occurrence of false-negative diagnoses, which may delay reperfusion therapy and adversely affect clinical outcomes. In contrast, false-positive STEMI diagnoses primarily result in unnecessary catheterization laboratory activation, increased resource utilization, and potential exposure of patients to unnecessary invasive procedures. With an overall sensitivity of approximately 80%, prehospital ECG interpretation may fail to identify up to one in five STEMI cases during the initial assessment, particularly in patients presenting with LBBB, paced rhythm, or ECG changes that do not meet the classical ST-segment elevation criteria [19,23]. For this reason, guidelines emphasize the need to integrate ECG interpretation with the clinical picture and the availability of rapid specialist consultation [11]. Improving the accuracy of STEMI diagnosis in teletransmission systems directly translates into shorter time to reperfusion and improved survival rates. In a recent meta-analysis by Moxham et al., prehospital digital ECG transmission was associated with a 47% reduction in mortality compared with standard care without prehospital ECG transmission (OR 0.53, 95% CI 0.40–0.69; *p* < 0.001; I^2^ = 0%) [16]. However, these findings were derived predominantly from observational studies and should therefore be interpreted as demonstrating an association rather than a causal relationship. The effectiveness of STEMI diagnosis should therefore be defined as a balance between high sensitivity (minimizing missed diagnoses) and an acceptable rate of false activations, while maintaining the organizational efficiency of the myocardial infarction treatment system [2].

The most important task of ECG teletransmission is to improve treatment timing. Reducing delays in the diagnostic-therapeutic chain is crucial for prognosis in STEMI. Both the ESC 2023 and the 2025 ACC/AHA/ACEP/NAEMSP/SCAI guidelines emphasize the importance of minimizing total ischemic time through early diagnosis, rapid reperfusion, and coordinated regional systems of care. The ESC indicates that if the predicted time from diagnosis to PCI reperfusion exceeds 120 min, fibrinolysis should be considered as soon as possible (ideally within 10 min of diagnosis)—which underscores the importance of the earliest possible prehospital diagnosis [2,11].

Reduction in FMC–ECG time (the time from the first medical contact to the performance and interpretation of the ECG) is crucial. Telemedicine enables the performance of a 12-lead ECG at the point of FMC and its immediate interpretation (locally or remotely), which shifts the diagnosis of STEMI to the prehospital phase. From the perspective of guidelines, it is crucial that the “clock” for reperfusion delays starts at the moment the ECG is interpreted as STEMI (and not only after admission to the hospital) [2]. Although most studies primarily report the impact on times to reperfusion (rather than “FMC-ECG” as a separate endpoint), prehospital ECG with simultaneous teletransmission achieves a practical goal: diagnosing STEMI and activating the reperfusion network before the patient presents to the hospital [6,7].

Another key aspect of telemedicine is its ability to reduce FMC-to-device time (the interval from the first medical contact with healthcare professionals to guidewire crossing of the culprit lesion during primary PCI). The strongest evidence regarding the impact of prehospital ECG teletransmission on treatment delays comes from the meta-analysis by Moxham et al., which included 17 observational studies involving 4306 patients. Compared with standard care, prehospital digital ECG transmission was associated with a significant reduction in FMC-to-device time (mean difference −24.7 min) and door-to-device time (mean difference −33.3 min) [16]. Terkelsen et al. demonstrated that prehospital diagnosis, particularly when combined with direct referral to a PCI centre, significantly shortened the interval from ambulance dispatch to first balloon inflation by 41 min compared with initial diagnosis at a local hospital, and by as much as 81 min when patients were transported directly to a PCI centre [7]. Sejersten et al. showed that prehospital ECG transmission directly to a cardiologist, combined with bypassing the local hospital, reduced the door-to-PCI time by more than 60 min [6].

These data support the thesis that ECG teletransmission is a “time-saving” intervention with real clinical significance: it reduces both in-hospital delays (door-to-device) and the total FMC-to-device time.

Beyond improving organizational efficiency, shortening total ischemic time is also biologically relevant. Earlier restoration of coronary blood flow limits infarct size reduces microvascular injury, and may attenuate adverse left ventricular remodeling, all of which contribute to improved long-term clinical outcomes. Consequently, the benefits of prehospital ECG teletransmission extend beyond reducing treatment delays, as timely reperfusion represents one of the fundamental components of contemporary cardioprotective strategies in STEMI [25].

Teletransmission thus enables the initiation of organizational procedures even before the patient’s arrival: remote consultation, PCI eligibility assessment, and early activation of the hemodynamic team. In the classic model described by Sejersten et al., prehospital ECG transmission to a cardiologist facilitated direct referral of the patient to a PCI center, bypassing the local hospital; in practice, this effect is a consequence of an earlier decision and the readiness of the catheterization laboratory to admit the patient [7].

Data from regional systems and implementation experiences indicate that teletransmission and teleconsultation are organizational elements that enable a streamlined “direct-to-PCI” pathway (by limiting unnecessary intermediate steps), which directly translates into shorter times to reperfusion [7,14,15]. At the same time, prehospital activation systems must balance time-related benefits against the risk of false-positive catheterization laboratory activations. A meta-analysis by Alrawashdeh et al. reported a false-positive rate of approximately 11% (depending on the interpretation model), which constitutes a measurable organizational “cost,” but one that is often accepted in exchange for shorter reperfusion times in patients with true STEMI [20]. Although the clinical value of ECG teletransmission is best established in STEMI, its potential role extends beyond immediate reperfusion pathways and also includes selected patients presenting with NSTE-ACS.

## 8. The Use of Remote ECG Teletransmission Beyond STEMI

The teletransmission of a 12-lead ECG is most strongly indicated in STEMI; however, its role in diagnosis of NSTEMI is significant from the perspective of early risk stratification and the organization of the treatment pathway. In NSTEMI, dynamic ST/T changes, integration with high-sensitivity troponin results, and clinical assessment are of key importance, as they determine the urgency of an invasive strategy. In contrast to STEMI, where ST-segment elevation is a diagnostic criterion, in NSTEMI, the ECG often shows ST-segment depression, T-wave inversion, and nonspecific repolarization changes and, in some cases, remains normal. The ESC guidelines for NSTE-ACS emphasize that dynamic, new, or likely new ST/T changes are a marker of high risk and constitute an indication for an early (<24 h) invasive strategy [2]. ECG teletransmission in NSTEMI allows for the comparison of serial recordings (e.g., during a recurrence of pain) and earlier detection of dynamic changes.

Unlike STEMI, the diagnosis of non-ST-segment elevation acute coronary syndrome (NSTE-ACS) cannot be established on the basis of ECG findings alone. Although prehospital ECG teletransmission may facilitate early identification of high-risk patients presenting with dynamic ST-segment or T-wave changes, recurrent ischemic symptoms, or hemodynamic instability, definitive diagnosis of NSTEMI requires integration of the clinical presentation with serial high-sensitivity cardiac troponin (hs-cTn) measurements. Current ESC and ACC/AHA guidelines recommend combining ECG findings with validated hs-cTn algorithms and formal risk assessment, including the GRACE risk score, to guide patient triage and determine the timing of an invasive strategy. Therefore, in patients with suspected NSTE-ACS, ECG teletransmission should primarily be regarded as a tool supporting early risk stratification and appropriate referral rather than as a definitive diagnostic modality.

In high-risk patients identified on the basis of the clinical presentation, dynamic ECG abnormalities, elevated hs-cTn concentrations, or a high GRACE risk score, an early invasive strategy is recommended according to current ESC and ACC/AHA guidelines. In this context, prehospital ECG teletransmission may facilitate earlier recognition of high-risk features, expedite referral to an appropriate cardiac centre, and improve coordination of subsequent in-hospital management.

A systematic review on prehospital ECG in the presence of chest pain showed that early acquisition and transmission of the recording influences the organization of care and may improve clinical outcomes even outside of STEMI [12].

According to the Fourth Universal Definition of Myocardial Infarction, the diagnosis of NSTEMI requires an increase or decrease in troponin levels accompanied by signs of ischemia (symptoms, ECG changes, imaging) [26]. Therefore, ECG teletransmission alone does not allow for a definitive diagnosis of NSTEMI but serves as a tool to aid in risk stratification. Additionally, variability in interpretation in the prehospital setting may influence a patient’s eligibility for urgent invasive diagnostics [13].

ECG teletransmission in patients with suspected NSTE-ACS therefore serves primarily as a triage and risk stratification tool rather than a definitive diagnostic modality; final decisions regarding invasive coronary angiography require integration of the clinical presentation, serial hs-cTn measurements, and formal risk assessment [2,26]. ECG teletransmission should not be performed routinely on all patients presenting with nonspecific symptoms, as it may lead to an overload of the consultation system and reduce the effectiveness of prehospital diagnostics. It is currently emphasized that the primary indication for ECG teletransmission should be a justified suspicion of acute coronary syndrome based on the clinical presentation or the presence of new abnormalities in the ECG recording.

Beyond acute coronary syndromes, telemedicine has become an integral component of contemporary cardiovascular care. Current applications include remote monitoring of patients with heart failure, telemonitoring of cardiac implantable electronic devices, cardiac rehabilitation, management of atrial fibrillation, and outpatient specialist consultations. Recent position papers emphasize that these technologies improve continuity of care, facilitate earlier detection of clinical deterioration, reduce unnecessary hospital visits, and support more personalized cardiovascular management, highlighting the growing role of digital health across multiple fields of cardiology [27].

## 9. Limitations of ECG Teletransmission

The quality of a prehospital ECG recording may be compromised by

- Patient movement (tremors, transport);
- Poor contact between the electrodes and the patient’s body;
- Incorrect electrode placement;
- Electrical interference.

Analyses of misdiagnosed STEMI indicate that ECG artifacts, incorrect electrode placement, conduction abnormalities (particularly LBBB), paced rhythm, and early repolarization are among the most common causes of diagnostic misclassification in the prehospital setting [23].

In NSTEMI, this issue is even more significant because ST/T changes are often subtle and dynamic, and a single recording may not show any abnormalities [2]. Furthermore, the effectiveness of ECG teletransmission is strictly dependent on the competence of the person interpreting the recording. In prehospital settings, significant variability in interpretation has been observed, which influenced triage decisions [12]. Diagnoses suggested by the teletransmission device often serve as a support for EMS teams. However, although computer algorithms achieve high specificity, their sensitivity is sometimes lower than that of clinical interpretation, which may increase the rate of false-negative diagnoses [18,19].

Teletransmission may be inappropriate in patients with established, previously diagnosed electrocardiographic changes, such as left bundle branch block, signs of left ventricular hypertrophy, or post-infarction changes, if there are no new symptoms suggesting myocardial ischemia. Examples of situations with limited diagnostic value include teletransmissions performed in patients with isolated hypertension, chronic and nonspecific chest pain, abdominal pain without signs of ischemia, syncope without ischemic changes on the ECG, or palpitations with a normal electrocardiogram. Overuse of teletransmission systems in low-risk cases may prolong the evaluation time for recordings requiring urgent cardiac intervention and increase the workload on consulting staff [5]. Technical issues often limit the use of telemedicine in cardiology. This is because ECG teletransmission requires a stable network connection, compatibility between IT (information technology) systems, and adequate security for medical data. In practice, transmission delays, signal loss, or interoperability issues between devices from different manufacturers may occur. Cloud-based solutions and wireless transmission systems, while increasing data accessibility, generate additional requirements regarding cybersecurity and personal data protection [28].

Although mobile network coverage in Poland is generally high, approximately 8% of the national territory remains outside reliable mobile network coverage, which may limit the implementation of ECG teletransmission in selected rural regions [28]. Similar challenges are observed globally. According to the International Telecommunication Union (ITU), while fourth-generation (4G) mobile networks cover approximately 92% of the world’s population, coverage remains substantially lower in rural areas (82%) than in urban areas (99%), and approximately 4% of the global population still lives beyond the reach of any mobile broadband network. These disparities may represent an important barrier to the implementation of telemedicine services, particularly in geographically underserved regions [29].

These disparities may contribute to unequal access to timely STEMI diagnosis and specialist consultation, representing an important health-equity challenge for the implementation of telecardiology systems.

Currently, there are many projects underway aimed at developing broadband internet and digitizing rural areas, funded by the European Union, such as the Digital Poland Operational Program (POPC) [30] and the European Funds for Digital Development (EFDC) [31]. In terms of infrastructure development, these projects primarily involve the construction of fiber-optic networks. Due to the objectives of these projects, mobile phone base stations, cell towers, and components of the wireless access network (LTE/5G) are not funded under these programs. Therefore, as a result of these development projects, the problem of insufficient wireless network coverage to ensure the proper functioning of telecommunications systems will persist. When establishing a telemedicine network, the main constraint is the cost of implementation, which involves the purchase of transmission-capable equipment, the development of IT infrastructure, staff training, system maintenance, and technical support. Although organizational analyses indicate that prehospital teletransmission systems improve the effectiveness of STEMI treatment and reduce delays [14], the direct costs of implementation may pose a barrier for smaller centers or systems with limited resources. In the long term, however, the potential benefits (reduced mortality, shorter hospital stays, and optimized use of the catheterization laboratories) may offset the investment costs [16].

## 10. Limitations of the Review

This review has several methodological limitations that should be acknowledged. First, it was conducted as a narrative review; therefore, a systematic review methodology (e.g., PRISMA) was not applied. Second, the literature search was performed using a single bibliographic database (PubMed), which may have resulted in the omission of relevant studies indexed elsewhere. Third, no formal assessment of study quality or risk of bias was performed, as the aim of this review was to provide a narrative synthesis rather than a quantitative evidence appraisal. Finally, the currently available evidence on prehospital ECG teletransmission is derived predominantly from observational studies; therefore, conclusions regarding clinical outcomes, particularly mortality, should be interpreted with appropriate caution. Despite these limitations, the narrative review methodology was considered appropriate for integrating evidence from clinical studies, guideline documents, and organizational aspects of regional STEMI systems of care, which are difficult to synthesize using a strictly systematic review methodology.

## 11. The Evolution of ECG Teletransmission Systems

Building on the current organization of prehospital ECG teletransmission systems, emerging digital technologies and artificial intelligence (AI) may further improve diagnostic support and workflow efficiency in the future. Future developments are expected to include more advanced AI algorithms, automated ECG interpretation, full integration with emergency medical services (EMS), and broader implementation of telecardiology consultations. Modern machine-learning and deep-learning algorithms enable the analysis of raw ECG signals beyond conventional amplitude- and time-based criteria. AI-assisted ECG interpretation has shown the potential to identify subtle electrocardiographic patterns of myocardial ischemia and may facilitate earlier recognition of STEMI in selected patients before all conventional ECG criteria become fully apparent. A recent retrospective cohort study evaluating AI-assisted prehospital ECG interpretation demonstrated the potential to reduce false-positive catheterization laboratory activations while maintaining high diagnostic sensitivity [22]. Although these findings are encouraging, they originate from a single retrospective cohort study and therefore require confirmation in prospective multicentre investigations before widespread clinical implementation. At the same time, previous analyses of the limitations of computer-aided interpretation emphasize that algorithms—despite increasing precision—are not error-free and require clinical oversight [18].

Despite these promising developments, the implementation of artificial intelligence in prehospital ECG interpretation requires careful clinical validation before widespread adoption. Most currently available algorithms have been evaluated using retrospective datasets, and prospective multicentre external validation across different emergency medical services (EMS) systems remains necessary to confirm their generalizability and diagnostic performance. Differences in patient populations, ECG acquisition methods, signal quality, and local clinical workflows may lead to dataset shift, potentially reducing algorithm performance outside the original development environment. In addition, false-negative AI classifications may delay reperfusion therapy, while ECG artifacts and motion-related noise remain important technical challenges that algorithms must reliably address.

Furthermore, many deep-learning models function as “black-box” systems, limiting the explainability of individual diagnostic decisions. This may affect clinician confidence, regulatory approval, and medicolegal responsibility for AI-assisted decision-making. Therefore, AI should currently be regarded as a clinical decision-support tool rather than a replacement for physician or paramedic judgement. Maintaining appropriate human oversight is essential to minimize automation bias and to ensure the safe integration of AI into routine prehospital workflows. Similar challenges have been highlighted in recent reviews of artificial intelligence in cardiovascular care, emphasizing that robust clinical validation, transparency, and integration into existing clinical pathways are prerequisites for widespread implementation [32]. Taken together, these considerations indicate that artificial intelligence should complement, rather than replace, clinical judgement in the prehospital setting. Successful implementation will depend not only on algorithm performance but also on rigorous clinical validation, integration into existing care pathways, and continued physician oversight.

Future developments may include predictive models integrating ECG findings, vital signs, and clinical data to support real-time risk stratification in the ambulance. However, such multimodal decision-support systems have not yet been prospectively validated and should currently be regarded as promising research concepts rather than established clinical tools.

Another area of development is the full integration of ECG teletransmission with EMS management systems. This includes the automatic transmission of ECG recordings to a referral centre, simultaneous transfer of clinical data (e.g., blood pressure, oxygen saturation, and heart rate), and integration with location and logistics systems to facilitate selection of the most appropriate PCI centre. In the future, EMS systems may also use predictive algorithms to dynamically select the destination hospital based on catheterization laboratory availability, transport time, and the patient’s estimated clinical risk.

## 12. Conclusions

Prehospital 12-lead ECG teletransmission has become a key component of contemporary systems of care for patients with acute coronary syndromes. It enables the diagnosis of STEMI to be made at the prehospital stage, allowing for earlier initiation of the reperfusion pathway and optimization of treatment logistics. Data from observational studies and meta-analyses clearly indicate that the implementation of prehospital ECG teletransmission is associated with a significant reduction in FMC–device and door-to-device times, which translates into improved patient outcomes. Prehospital ECG teletransmission has been associated with a 47% relative reduction in mortality in observational evidence, although causality cannot be definitively established [16].

At the same time, teletransmission plays a significant role in risk stratification of patients with NSTEMI, supporting decisions regarding the urgency of invasive strategies and the selection of patients for referral to specialized centers. The integration of this technology with EMS systems and the development of cardiac teleconsultations strengthen the organizational aspect of this intervention.

Despite limitations—such as the risk of false activations of the catheterization laboratory, dependence on the quality of the recording, and the experience of the interpreter—the balance of clinical and systemic benefits supports treating ECG teletransmission as the standard in integrated myocardial infarction treatment systems. The further development of AI algorithms and the full digitization of the care pathway may further enhance the effectiveness and safety of this model in the future.

Ultimately, the clinical value of prehospital ECG teletransmission depends not only on the technology itself but also on its successful integration within coordinated regional STEMI systems of care, supported by appropriate EMS training, standardized ECG interpretation pathways, timely catheterization laboratory activation, regional PCI availability, and continuous quality improvement.

## Figures and Tables

**Table 1:** Comparison of Store-and-Forward and Real-Time Transmission Telecardiology Systems.

Feature	Real-Time Transmission	Store-and-Forward
Data transmission time	Immediate	Delayed
Physician interaction	Yes	Usually no
Application in STEMI	Critical	Limited
Required infrastructure	Dedicated transmission link	Lower requirements

**Table 2:** Landmark clinical studies and meta-analyses evaluating prehospital ECG teletransmission in acute coronary syndromes.

Study/Year	Design/Population	ECG Transmission Model	Comparator	Main Findings	Limitations
Sejersten et al. [6]	Prospective observational study; STEMI patients	Prehospital ECG teletransmission to cardiologist with direct PCI referral	Standard hospital pathway	Door-to-PCI time reduced by >60 min; facilitated direct transfer to PCI centre	Single regional network; observational design
Terkelsen et al. [7]	Observational cohort; STEMI patients	Prehospital diagnosis with direct referral to PCI centre	Diagnosis at local hospital	Ambulance dispatch-to-balloon time reduced by 41–81 min depending on transport strategy	Non-randomized study; regional organization
Nakashima et al. [12]	Systematic review	Prehospital 12-lead ECG with hospital notification	Standard prehospital care	Associated with lower mortality and shorter treatment delays	Predominantly observational studies; heterogeneity among included studies
Moxham et al. [16]	Systematic review and meta-analysis (17 studies; 4306 patients)	Prehospital digital ECG transmission	Standard care without ECG transmission	FMC-to-device time −24.7 min; door-to-device time −33.3 min; associated with lower mortality (OR 0.53, 95% CI 0.40–0.69)	Included studies observational; heterogeneity in time-related outcomes
Alrawashdeh et al. [19]	Systematic review and meta-analysis (>67,000 patients)	Ambulance clinician, computer-assisted and remote specialist ECG interpretation	Comparison of ECG interpretation strategies	Sensitivity 80%, specificity 97%; false-positive cath-lab activation ~11–16%	High between-study heterogeneity; variability of interpretation models
Tanguay et al. [20]	Observational study	Wireless ECG transmission with direct cath-lab activation	Standard referral pathway	Improved appropriateness of primary PCI activation and reduced treatment delays	Single healthcare system
Tanguay et al. [21]	Diagnostic accuracy study	Remote ECG interpretation by emergency physicians	Conventional ECG interpretation	High agreement with coronary angiography while reducing unnecessary cath-lab activation	Single-centre experience
Baker et al. [22]	Retrospective cohort study	AI-assisted prehospital ECG interpretation	Conventional ECG interpretation	Reduced false-positive catheterization laboratory activations while maintaining high diagnostic sensitivity	Retrospective design; external validation required

## Data Availability

No new data were created or analyzed in this study. Data sharing is not applicable to this article.

## References

[1] Timmis A., Townsend N., Gale C.P., Torbica A., Lettino M., Petersen S.E., Mossialos E.A., Maggioni A.P., Kazakiewicz D., May H.T. (2020). European Society of Cardiology: Cardiovascular disease statistics 2019. Eur. Heart J..

[2] Byrne R.A., Rossello X., Coughlan J.J., Barbato E., Berry C., Chieffo A., Claeys M.J., Dan G.-A., Dweck M.R., Galbraith M. (2023). 2023 ESC Guidelines for the Management of Acute Coronary Syndromes. Eur. Heart J..

[3] Zuin M., Rigatelli G., Temporelli P., Di Fusco S.A., Colivicchi F., Pasquetto G., Bilato C. (2023). Trends in Acute Myocardial Infarction Mortality in the European Union, 2012–2020. Eur. J. Prev. Cardiol..

[4] Gawinski L.P., Kozlowski R., Mikulski J. (2020). Management of Processes of the Diagnosis and Treatment of Acute Myocardial Infarction Using Telematics Systems. Research and the Future of Telematics.

[5] Gawinski L., Burzynska M., Kamecka K., Kozlowski R. (2022). Practical Aspects of the Use of Telematic Systems in the Diagnosis of Acute Coronary Syndrome in Poland. Medicina.

[6] Sejersten M., Sillesen M., Hansen P.R., Nielsen S.L., Nielsen H., Trautner S., Hampton D., Wagner G.S., Clemmensen P. (2008). Effect on Treatment Delay of Pre-Hospital Teletransmission of 12-Lead Electrocardiogram to a Cardiologist for Immediate Triage and Direct Referral of Patients with ST-Segment Elevation Acute Myocardial Infarction to Primary Percutaneous Coronary Intervention. Am. J. Cardiol..

[7] Terkelsen C.J., Lassen J.F., Nørgaard B.L., Gerdes J.C., Poulsen S.H., Bendix K., Ankersen J.P., Gøtzsche L.B.-H., Rømer F.K., Nielsen T.T. (2005). Reduction of Treatment Delay in Patients with ST-Elevation Myocardial Infarction: Impact of Pre-Hospital Diagnosis and Direct Referral to Percutaneous Coronary Intervention. Eur. Heart J..

[8] Andersen H.R., Nielsen T.T., Rasmussen K., Thuesen L., Kelbaek H., Thayssen P., Abildgaard U., Pedersen F., Madsen J.K., Grande P. (2003). A Comparison of Coronary Angioplasty with Fibrinolytic Therapy in Acute Myocardial Infarction (DANAMI-2). N. Engl. J. Med..

[9] Diercks D.B., Kontos M.C., Chen A.Y., Pollack C.V., Wiviott S.D., Rumsfeld J.S., Magid D.J., Gibler W.B., Cannon C.P., Peterson E.D. (2009). Utilization and Impact of Prehospital ECG in Patients with STEMI. J. Am. Coll. Cardiol..

[10] Widimsky P., Budesinsky T., Vorac D., Groch L., Želízko M., Aschermann M., Branny M., Štásek J., Formánek P., ‘PRAGUE’ Study Group Investigators (2000). Long Distance Transport for Primary Angioplasty vs. Immediate Thrombolysis (PRAGUE Study). Eur. Heart J..

[11] Rao S.V., O’Donoghue M.L., Ruel M., Rab T., Tamis-Holland J.E., Alexander J.H., Baber U., Baker H., Cohen M.G., Cruz-Ruiz M. (2025). 2025 ACC/AHA/ACEP/NAEMSP/SCAI Guideline for the Management of Patients with Acute Coronary Syndromes. J. Am. Coll. Cardiol..

[12] Nakashima T., Hashiba K., Kikuchi M., Yamaguchi J., Kojima S., Hanada H., Mano T., Yamamoto T., Tanaka A., Matsuo K. (2022). Impact of Prehospital 12-Lead Electrocardiography and Destination Hospital Notification on Mortality in Patients with Chest Pain: A Systematic Review. Circ. Rep..

[13] Kudenchuk P.J., Maynard C., Cobb L.A., Wirkus M., Martin J.S., Kennedy J.W., Weaver W.D. (1998). Utility of the Prehospital Electrocardiogram in Diagnosing Acute Coronary Syndromes: The Myocardial Infarction Triage and Intervention (MITI) Project. J. Am. Coll. Cardiol..

[14] Meloni L., Floris R., Montisci R., De Candia G., Cadeddu M., Lai G., Sori P., Ruscazio M., Pinna G., Iasiello G. (2016). Care Quality Monitoring of a ST-Segment Elevation Myocardial Infarction Programme over a 5-Year Period. J. Cardiovasc. Med..

[15] Kleinrok A., Płaczkiewicz D.T., Puźniak M., Dąbrowski P., Adamczyk T. (2014). Electrocardiogram Teletransmission and Teleconsultation: Essential Elements of the Organisation of Medical Care for Patients with ST-Segment Elevation Myocardial Infarction: A Single Centre Experience. Kardiol. Pol..

[16] Moxham R.N., D’eNtremont M.-A., Mir H., Schwalm J., Natarajan M.K., Jolly S.S. (2024). Effect of Prehospital Digital Electrocardiogram Transmission on Revascularization Delays and Mortality in ST-Elevation Myocardial Infarction Patients: Systematic Review and Meta-Analysis. CJC Open.

[17] Al-Zaiti S.S., Shusterman V., Carey M.G. (2013). Novel Technical Solutions for Wireless ECG Transmission and Analysis in the Age of the Internet Cloud. J. Electrocardiol..

[18] Schläpfer J., Wellens H.J.J. (2017). Computer-Interpreted Electrocardiograms: Benefits and Limitations. J. Am. Coll. Cardiol..

[19] Alrawashdeh A., Ihtoub S., Alkhatib Z.I., Alwidyan M., Khader Y.S., Rawashdeh S., Alqahtani S., Stub D., Alhamouri R., Alkhazali I.E. (2025). Prehospital ECG Interpretation Methods for ST-Elevation Myocardial Infarction Detection and Catheterization Laboratory Activation: A Systematic Review and Meta-Analysis. Arch. Acad. Emerg. Med..

[20] Tanguay A., Lebon J., Brassard E., Hébert D., Bégin F. (2019). Diagnostic Accuracy of Prehospital Electrocardiograms Interpreted Remotely by Emergency Physicians in Myocardial Infarction Patients. Am. J. Emerg. Med..

[21] Tanguay A., Brassard E., Lebon J., Bégin F., Hébert D., Paradis J.-M. (2017). Effectiveness of a Prehospital Wireless 12-Lead Electrocardiogram and Cardiac Catheterization Laboratory Activation for ST-Elevation Myocardial Infarction. Am. J. Cardiol..

[22] Baker P.O., Karim S.R., Smith S.W., Meyers H.P., Robinson A.E., Ibtida I., Karim R.M., Keller G.A., Royce K.A., Puskarich M.A. (2025). Artificial Intelligence Driven Prehospital ECG Interpretation for the Reduction of False Positive Emergent Cardiac Catheterization Lab Activations: A Retrospective Cohort Study. Prehosp. Emerg. Care.

[23] Bosson N., Sanko S., Stickney R.E., Niemann J., French W.J., Jollis J.G., Kontos M.C., Taylor T.G., Macfarlane P.W., Tadeo R. (2017). Causes of Prehospital Misinterpretations of ST-Elevation Myocardial Infarction. Prehosp. Emerg. Care.

[24] Coffey C., Serra J., Goebel M., Espinoza S., Castillo E., Dunford J. (2018). Prehospital Acute ST-Elevation Myocardial Infarction Identification in San Diego: A Retrospective Analysis of the Effect of a New Software Algorithm. J. Emerg. Med..

[25] Grigoriou K., Karakasis P., Lamprou V., Michas G., Pamporis K., Trikas A., Pantos C., Mourouzis I. (2025). Cardioprotective Therapies for ST-Elevation Myocardial Infarction: The Emerging Role of Thyroid Hormone: A Narrative Review. Front. Endocrinol..

[26] Thygesen K., Alpert J.S., Jaffe A.S., Chaitman B.R., Bax J.J., Morrow D.A., White H.D. (2019). Fourth Universal Definition of Myocardial Infarction (2018). Eur. Heart J..

[27] Pierucci N., Laviola D., Mariani M.V., Nardini A., Adamo F., Mahfouz K., Colaiaco C., Ammirati F., Santini L., Lavalle C. (2025). Remote monitoring and heart failure. Eur. Heart J. Suppl..

[28] Office of Electronic Communication (2025). Report on the State of the Telecommunications Market 2024.

[29] More than Half of the World’s Population Now Covered by 5G. https://www.itu.int/itu-d/reports/statistics/2024/11/10/ff24-mobile-network-coverage.

[30] Ministry of Funds and Regional Policy (2025). Digital Poland Programme.

[31] Government of Poland (2025). European Funds for Digital Development—Digital Poland Project Center.

[32] Karakasis P., Theofilis P., Sagris M., Pamporis K., Stachteas P., Sidiropoulos G., Vlachakis P.K., Patoulias D., Antoniadis A.P., Fragakis N. (2025). Artificial Intelligence in Atrial Fibrillation: From Early Detection to Precision Therapy. J. Clin. Med..

